# Open-Spaced Ridged Hydrogel Scaffolds Containing TiO_2_-Self-Assembled Monolayer of Phosphonates Promote Regeneration and Recovery Following Spinal Cord Injury

**DOI:** 10.3390/ijms241210250

**Published:** 2023-06-16

**Authors:** Ahad M. Siddiqui, Frederic Thiele, Rachel N. Stewart, Simone Rangnick, Georgina J. Weiss, Bingkun K. Chen, Jodi L. Silvernail, Tammy Strickland, Jarred J. Nesbitt, Kelly Lim, Jean E. Schwarzbauer, Jeffrey Schwartz, Michael J. Yaszemski, Anthony J. Windebank, Nicolas N. Madigan

**Affiliations:** 1 Department of Neurology, Mayo Clinic, Rochester, MN 55905, USA; 2 Program in Human Medicine, Paracelsus Medical Private University, 5020 Salzburg, Austria; 3 Regenerative Medicine Institute (REMEDI), National University of Ireland Galway, H91 TK33 Galway, Ireland; 4 Program in Human Medicine, Paracelsus Medical Private University, 90419 Nuremberg, Germany; 5 Department of Chemistry, Princeton University, Princeton, NJ 08544, USA; 6 Department of Molecular Biology, Princeton University, Princeton, NJ 08544, USA; 7 Department of Orthopedic Surgery, Mayo Clinic, Rochester, MN 55905, USA

**Keywords:** axon regeneration, biomaterials, immune cells, machine learning, mesenchymal stromal cells (MSCs), oligo(poly(ethylene glycol)) fumarate, Schwann cells, spinal cord injury, titanium dioxide, scarring

## Abstract

The spinal cord has a poor ability to regenerate after an injury, which may be due to cell loss, cyst formation, inflammation, and scarring. A promising approach to treating a spinal cord injury (SCI) is the use of biomaterials. We have developed a novel hydrogel scaffold fabricated from oligo(poly(ethylene glycol) fumarate) (OPF) as a 0.08 mm thick sheet containing polymer ridges and a cell-attractive surface on the other side. When the cells are cultured on OPF via chemical patterning, the cells attach, align, and deposit ECM along the direction of the pattern. Animals implanted with the rolled scaffold sheets had greater hindlimb recovery compared to that of the multichannel scaffold control, which is likely due to the greater number of axons growing across it. The immune cell number (microglia or hemopoietic cells: 50–120 cells/mm^2^ in all conditions), scarring (5–10% in all conditions), and ECM deposits (Laminin or Fibronectin: approximately 10–20% in all conditions) were equal in all conditions. Overall, the results suggest that the scaffold sheets promote axon outgrowth that can be guided across the scaffold, thereby promoting hindlimb recovery. This study provides a hydrogel scaffold construct that can be used in vitro for cell characterization or in vivo for future neuroprosthetics, devices, or cell and ECM delivery.

## 1. Introduction 

The utilization of tissue engineering and material science in regenerative medicine for the treatment of a spinal cord injury (SCI) presents an opportunity to modify the hostile microenvironment to promote regeneration after the injury. Traumatic SCIs occur due to an insult of the vertebral column, resulting in the contusion, compression, laceration, or transection of the spinal cord. This primary injury leads to a host of secondary injury events, such as inflammation, cell loss, scarring, and cavitation, which not only create a hostile microenvironment, but a physical gap that impedes regeneration [1]. Hydrogels can be constructed into scaffolding for axons to regenerate across, thereby bridging the injury. Physical and chemical configurations of the scaffold are important factors to consider when developing new designs and biomaterials. They should match the existing spinal cord environment and hinder the regeneration as little as possible. Attributes such as stiffness, permeability, swelling, strength, and degradation kinetics are the most important characteristics, which define the reaction of the surrounding tissue to the foreign implant [2]. In addition, the physical characteristics of scaffolds can change due to degradation and physiological stressors, which are ideally tested in vivo [3]. The macro- and micro-architecture of the scaffold can greatly affect its ability to promote regeneration and recovery following an SCI [4]. Many considerations in the type of material or design play an important role in balancing the effects of regeneration verses inflammation or scarring because of the introduction of a foreign object. 

**Figure 1:**
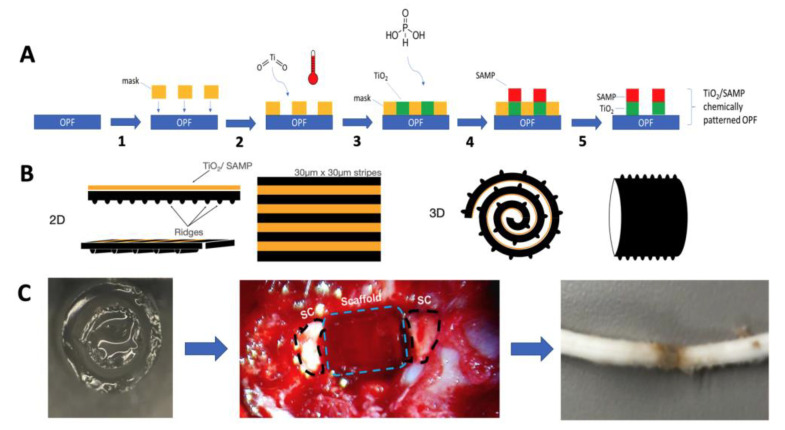
Summary of the methodology of chemical patterning on OPF scaffolds and implantation of the rolled scaffolds. (**A**) Schematic for debossing OPF with the micron-scale chemical pattern. The shadow mask was placed onto the OPF surface (1). The substrate was exposed to titanium dioxide and heat (2), forming a cross-linked TiO_2_ base layer. Overnight exposure to phosphonic acid (3) yielded the self-assembled layer of phosphonate (4). The mask was removed to generate 30 μm × 30 μm TiO_2_/SAMP pattern on the polymer surface (5). (**B**) The resulting OPF sheet consisted of a ridged and chemically patterned surface. The sheet could then be rolled with the ridges, which also act as a spacer. (**C**) Once rolled, the sheet was placed in a single channel OPF scaffold to hold the structure together from implantation into the transected thoracic level 9 spinal cord. The spinal cord, when it has been removed, shows integration with the spinal cord tissue.

Several scaffold macro-architectures designs have been investigated. These designs fall into five broad categories consisting of a spongey cylinder, single tube, channels, an open path with a core, and an open path without core [5]. Open-path scaffolds can increase axon regeneration, while maintaining the defect size, while other scaffold designs do not show as much axon growth and have an increased defect size [5]. However, other studies have demonstrated that multichannel designs can aid axon regeneration and have reduced cysts sizes, and they have been designed to match the spatial distribution of spinal cord tracts [6,7]. Another influence on axon regeneration is the diameter of the channels, where often reducing the channel size can increase the axon count [8]. For example, reducing the channel size from 660 μm to 450 μm in diameter nearly doubled the number of axons that grew [8]. Channels as small as 200 μm have been shown to promote axon regeneration, and the in vitro characterization of astrocytes shows that smaller-diameter scaffolds alter alignment and proliferation [9,10]. Another strategy to improve alignment involved using grooves or ridges to introduce orientation via methods such as lithography [11,12], molding [13,14], or 3D biofabrication [15,16]. Our group recently developed a method via which the surface topology could be added to a water-rich hydrogel via chemical processing utilizing hydrolysis [17]. This surface chemistry uses a coating of titanium dioxide (TiO_2_) to layer a surface of phosphonic acid to create a self-assembled monolayer of phosphonates (SAMP; TiSAMP) that is only a few molecular layers high and can be patterned to be 30 μm wide (Figure 1A). When it is patterned, the fibroblasts extend and deposit ECM along the direction of the pattern [18]. 

One concern with implantable biomaterials, particularly as further modifications are made, is the biocompatibility of the material to the host tissue. It has been often observed that in matter of weeks, implant materials cause foreign body reaction that results in the accumulation of collagenous capsule and fibrosis with low vascularity and inflammation [19,20,21]. One approach has been to reduce the area of the biomaterial available to interact with infiltrating cells or tissue; however, this often leads to fibrosis at the tissue–biomaterial interface [22,23]. Factors such as porosity, hydrophobicity, surface topography, stiffness, surface chemistry, and other active components can influence inflammation [24]. For example, the ability of biomaterials to bind damage-associated molecular patterns (DAMPs) released from injured tissues may lead to greater inflammation around the biomaterial due to recognition by immune cells. As the material is degraded, the DAMPs may re-release, leading macrophage activation and chronic inflammation [25,26]. Therefore, novel scaffold designs, modification, and biomaterials should be tested to ensure scarring or inflammation is not enhanced.

We have developed a hydrogel composed of oligo(poly(ethylene glycol)fumarate) (OPF) that is biodegradable, porous, and biomechanically similar to the spinal cord [27,28]. When it is fabricated in a multichannel design, it can reduce scarring and cyst formation and can induce greater axon outgrowth than poly(lactic-co-glycolic acid) (PLGA)- and poly(caprolactone fumarate) (PCLF)-based scaffolds can [28,29]. OPF can be easily utilized for combinatorial therapy via combining them with cells, drug eluting microspheres, extracellular matrix (ECM) proteins, or modifying the material with additional characteristics, such as a positive charge or improved electroconductivity [13,28,29,30,31,32,33,34,35]. All these modifications aim to improve regeneration and recovery following an SCI.

One important observation to come from these studies is that directing axon growth across the scaffold is important, and although the multichannel design helps achieve this, many of the axons still grow on the outside surface of the scaffold. In an aim to improve directed axon growth through the scaffold, we modified the design of the scaffold to introduce a greater internal surface area by fabricating the scaffold as a ridged sheet [13]. The ridges help to direct neurite growth in a bidirectional manner, and when they are placed closer together, they can increase the number of neurites per surface area [13]. However, there is a limit to how close ridges can be placed together using molding techniques. To overcome this obstacle, we created a process by which TiO_2_ can be used to layer SAMP on hydrogels within micrometers of each other [18]. This study is the first application of these rolled scaffolds containing TiSAMP in a rodent SCI model. Since in vivo cellular interactions with biomaterials are more complex than those that occur in vitro and these influences can affect axon regeneration, this is an important step toward possible future human use. This current study demonstrates that an open-design, ridged scaffold sheet via a cell-attractive TiSAMP chemical treatment (Figure 1) improves axon outgrowth further without increasing inflammation and scarring as compared to that of the more commonly used multichannel scaffold design. In addition, this study details a method with which machine learning algorithms can be used to analyze immune cell infiltration and scarring following scaffold implantation in a fast, non-biased manner. 

## 2. Results

### 2.1. Cultured Mesenchymal Stromal Cells form Spheres on Ridged Surfaces

**Figure 2:**
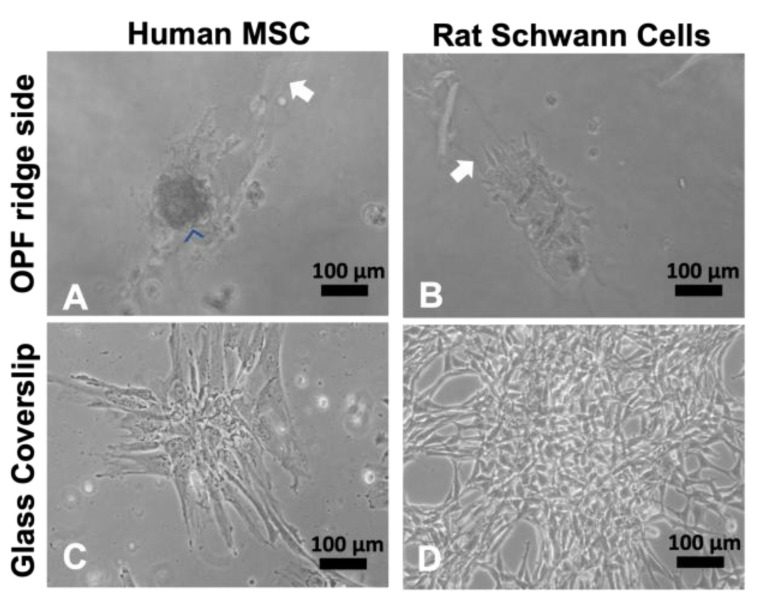
Human MSCs and rat Schwann cells grown on OPF+ scaffold sheets with ridges spaced 0.4 mm apart. Live culture images taken at 10× bright-field. (**A**) Human MSCs attach, but do not spread (blue arrowheads) when grown on ridged OPF+ sheets, whereas the rat Schwann cells (**B**) attach and spread along the ridges (raised portions of the polymer are indicated by a white arrow). This is not observed on glass coverslips where hMSCs (**C**) and Schwann cells (**D**) spread into their stereotypical morphologies.

OPF+ scaffold sheets were fabricated with 0.1 mm high ridges spaced 0.4 mm apart on one side. The other side contained a bare polymer. Our previous study demonstrated that ECM protein-coated, ridged OPF+ sheets promoted Schwann cell and neuronal alignment and outgrowth along the ridge in vitro [13]. However, different cell types respond differently to topographic cues (ridges or material stiffness); so, two commonly investigated cell types for SCI treatment (mesenchymal stromal cells (MSCs) and Schwann cells) were compared. Human MSC (hMSCs) and rat Schwann cells were grown on the ridged OPF+ sheet and glass coverslips (Figure 2A–D). At 3 days in the culture, the cells were found primarily on or near the ridges on the OPF+ sheet (Figure 2A,B; white arrows indicate ridges). The Schwann cells adhered and spread along the ridges (Figure 2B), while hMSCs did not spread, but instead adhered as large cell clusters (Figure 2A). In contrast, when they were grown on glass coverslips (Figure 2C,D), all the cell types exhibit their normal, stereotypical, well-spread, and flat shapes. 

**Figure 3:**
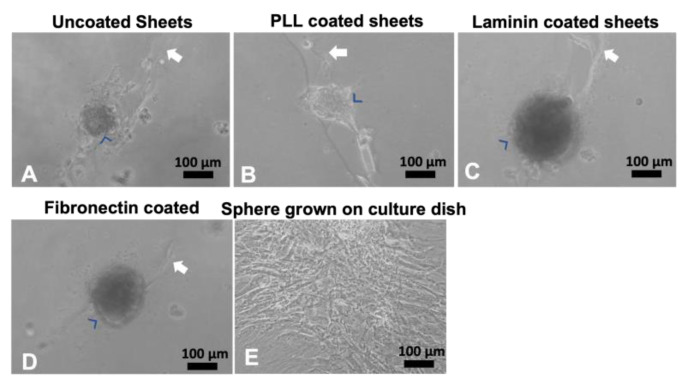
Human MSCs grown on coated OPF+ sheets containing ridges. Live culture images taken at 10x bright-field. Human MSCs adhered in cell clusters (blue arrowheads) when grown for 3 days on uncoated (**A**), poly-L-lysine-coated (**B**), laminin-coated (**C**), or fibronectin-coated (**D**) OPF+ ridged sheets (white arrow indicates the ridge). If these spheres were picked and grown in a culture dish, they spread into their stereotypical shape (**E**).

Since these OPF+ sheets were not coated with any ECM molecules other than those found in the media, we coated the OPF+ ridged sheets with PLL, laminin, or fibronectin protein coats. Again, we found that the hMSCs were found mostly on the ridges as cell clusters (Figure 3A–D) after 3 days in the culture. To test if these clusters were viable and exhibited a normal morphology, they were picked and plated in normal culture dishes (Figure 3E). The clusters were shown to spread on the culture dish and grow normally. 

**Figure 4:**
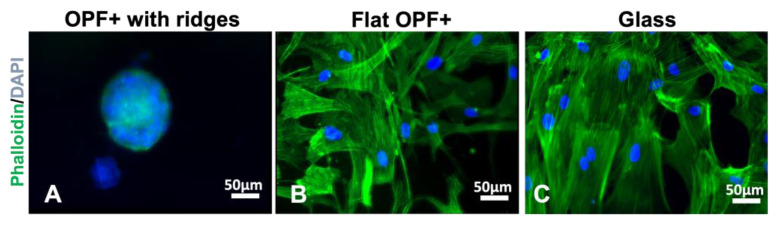
Staining for phalloidin and collagen IV of human MSCs cultured on OPF+ sheets with ridges, flat OPF+ sheets or glass. Human MSCs were grown for 7 days on OPF+ sheets containing ridges (**A**), on the flat side of the OPF+ sheet (**B**) or on glass (**C**), and then fixed, permeabilized, and stained with DAPI (blue) to visualize cell nuclei and phalloidin (green) for the actin cytoskeleton. On the ridged surface, multicellular clusters formed as indicated by multiple nuclei in a 3D spheroidal organization. On flat surfaces, cells attached and spread with a similar morphology.

Next, we tested if this behavior of the cells was due to material stiffness or the topography. The hMSC sphere contains multiple nucelli, and the phalloidin does not extend out on the material (Figure 4A). When hMSCs are grown on the flat side of the OPF+ scaffold, we found that the cells adhered to the material in a flat manner such as it does on glass and plastic (Figure 4B). Staining for actin with phalloidin, we show that the cytoskeletal structure looks like that of hMSCs grown on glass coverslips (Figure 4C). These results suggest that the 3D shape formed by the 0.1 mm high ridges induces cell clusters rather than the material being softer than plastic/glass is. 

### 2.2. Mesenchymal Stromal Cells, Schwann Cells, and DRG Neurons Attach to TiSAMP and Extend along the Chemical Pattern

**Figure 5:**
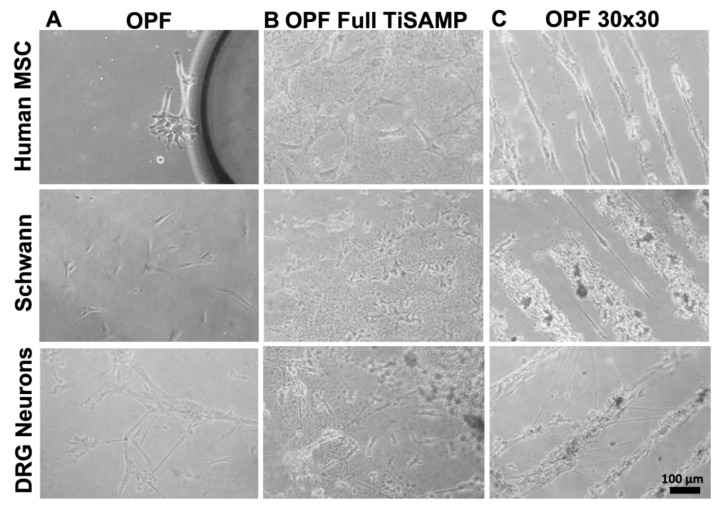
Attachment and alignment of human MSCs, Schwann cells, and dissociated DRG neurons on OPF scaffold sheets. Cells were plated on OPF scaffolds and grown for 5 days before staining and analysis. Cells on untreated OPF (**A**) showed no spatial directionality and were sparsely distributed in the culture after 2 days. (**B**) Cells on OPF scaffolds fully coated with TiSAMP (full TiSAMP) also demonstrated no spatial directionality, however, were more densely attached. (**C**) All 3 cell types aligned on chemically patterned OPF. DRG neurons sometimes branch out across the bare polymer to adjacent chemical strips that travel in a bidirectional orientation in the culture within 3 days.

**Figure 6:**
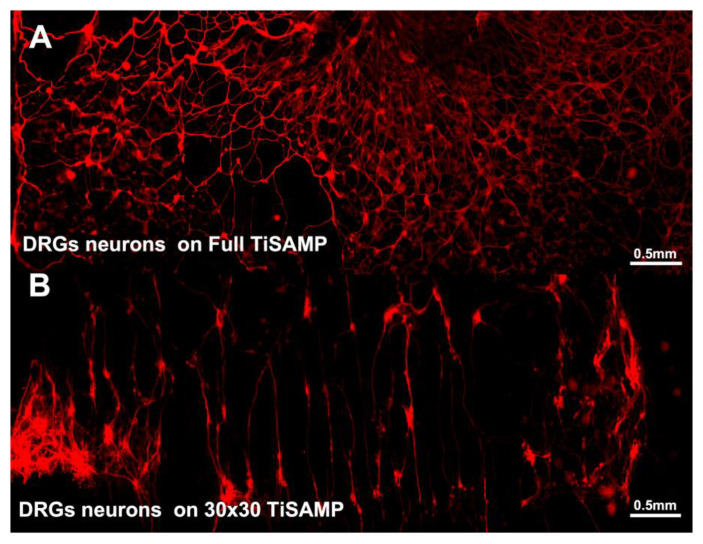
Tiled micrograph of immunohistochemical staining for β-tubulin of dispersed DRG neurons shows random distribution of neurons when it was plated on full TiSAMP, (**A**) but it was highly organized on the patterned 30 × 30 TiSAMP scaffold (**B**) in a bidirectional orientation. Some axons are seen on the adjust polymer area, but they often seen and oriented on a different chemical strip.

We fabricated ridged OPF scaffold sheets and deposited TiSAMP on the un-ridged side, which was either unpatterned (full TiSAMP) or patterned with 30 μm TiSAMP by 30 μm polymer (30 × 30 TiSAMP), using a shadow masking technique (Figure 1). The chemical processing of the OPF hydrogel scaffolds with TiSAMP has the advantages of being only a few molecular layers thick and can be patterned closer together than molding ridges can. The effect of the TiSAMP surface on outgrowth and ECM deposition by hMSCs, rat Schwann cells, and neurons was investigated. hMSCs, Schwann cells, and dispersed DRG neurons were plated on OPF scaffolds with no TiSAMP, full TiSAMP coating, and patterned 30 × 30 TiSAMP for 7 days. The cells adhered in a random orientation on the OPF (Figure 5A) and full TiSAMP (Figure 5B and Figure 6A), although the cells adhered more sparsely on the untreated OPF. On the OPF 30 × 30 TiSAMP, the cells aligned along the chemical pattern in a bidirectional fashion (Figure 5C). In some cases, the DRG neurons extended on the bare polymer, but then join at the contralateral pattern, still preferring the chemical’s bidirectional pattern, as detected via the anti-tubulin staining of neurites (Figure 5C and Figure 6B). These results demonstrate that neurite outgrowth is enhanced on the TiSAMP material, and alignment can be promoted via the striped pattern. Attachment and elongation are superior to that on the bare polymer, and therefore, may aid axon regeneration across the gap in the SCI animal model. 

**Figure 7:**
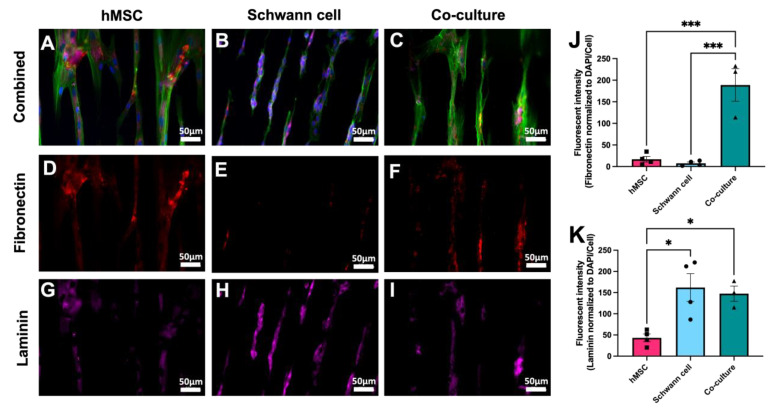
Attachment, polarization, and ECM deposit by hMSCs and rat Schwann cells on patterned 30 × 30 TiSAMP OPF. hMSCs (**A**) and Schwann cells (**B**) were cultured on the chemically patterned OPF individually and in co-culture (50:50) on the chemically patterned OPF for 7 days. Using immunocytochemistry, the scaffolds were stained for DAPI (blue), phalloidin (green; (**A**–**C**)), fibronectin (red, (**D**–**F**)) and laminin (pink, (**G**–**I**)) to compare ECM production. Fibronectin (**J**) and laminin (**K**) intensities were quantified using ImageJ via normalizing fibronectin production to DAPI/cell number (n = 4 scaffolds with 4 areas per sheet). Statistical analysis of fibronectin staining was run using a one-way ANOVA with multiple comparisons using Tukey’s post hoc test. The degrees of significance are indicated as follows: *p* < 0.05 (*) and *p* < 0.001 (***). Error bars represent standard error, individual data points are displayed for each bar.

**Figure 8:**
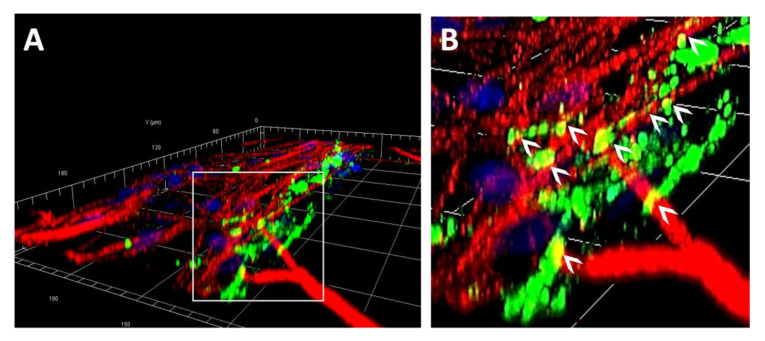
A 3D reconstruction of a confocal z-stack image is shown containing a grid, with each square representing 30 × 30 microns (**A**) and a zoomed-in section (**B**). Samples were stained for fibronectin (green), β-tubulin (neurons, red) and DAPI (cell nuclei, blue) after Schwann cells and neurons had been in culture for 10 and 4 days, respectively. Neurites were observed to weave over and through (white arrowheads) the network of the native, SC-specific ECM.

The different cell types may interact with the biomaterial via depositing ECM. hMSCs, Schwann cells, and a co-culture of hMSCs and Schwann cells were stained for fibronectin and laminin production. The co-cultured cells, such as the cells cultured alone did, aligned along the chemical pattern (Figure 7A–C). hMSCs produce more fibronectin alone than the Schwann cells did, although the difference was not statistically significant (Figure 7D,E; hMSC = 17.02 ± 6.43 mean gray value, and Schwann cells = 7.57 ± 2.87 mean gray value). The Schwann cells produced more laminin than hMSCs did (Figure 7G,H; hMSC = 43.33 ± 9.10 mean gray value, and Schwann cells = 161.9 ± 32.77 mean gray value, *p* = 0.013). The ECM deposits were in the orientation of chemical patterning. Co-cultures can deposit both ECM types (Figure 7F,I). Co-culturing hMSCs and Schwann cells resulted in greater fibronectin deposits (188.9 ± 37.67 mean gray value) than those produced using the Schwann cells (Figure 7J; *p* = 0.0004) or MSCs alone (*p* = 0.0003), suggesting that both cell types produce fibronectin. Co-culture staining for laminin was not significantly different from that of Schwann cells alone, but it was much better than using hMSCs alone (Figure 7K; hMSCs = 43.33 ± 9.10 mean gray value, co-culture = 147.50 ± 17.97 mean gray value, *p* = 0.0367) indicating that Schwann cells are primarily responsible for producing laminin in this system. DRG neurons were plated onto Schwann cell cultures, and DRG neurites were visualized via anti β-tubulin staining, which shows that some neurites extended along the fibronectin component of the ECM (Figure 8A,B). Together, these results indicate this TiSAMP-patterned material can support cell attachment, neurite outgrowth, and ECM deposition, all of which are important for SCI repair. 

### 2.3. Rolled Scaffolds Have Equal Number of Hemopoietic Cells, Microglia, and Other Cell Types as Multichannel Scaffolds

**Figure 9:**
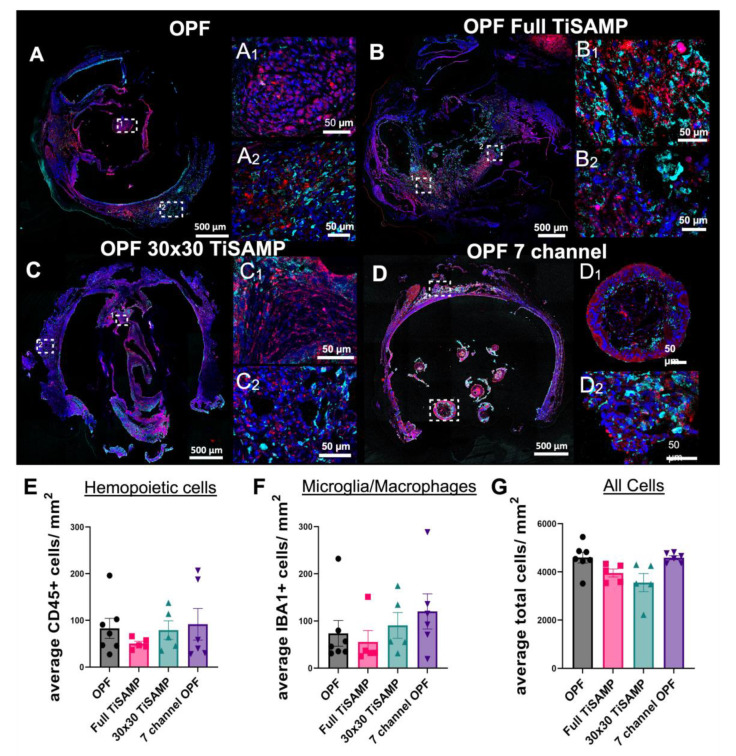
Infiltration of immune cells into the implant OPF scaffolds. Implanted untreated OPF (**A**), Full TiSAMP (**B**), 30 × 30 TiSAMP (**C**), and seven-channel (**D**) scaffold were sectioned and stained at quarter lengths (¼, ½, and ¾ of the scaffold; center or ½ segment shown here) for Iba-1 (red), CD45 (cyan), and DAPI (blue) to determine the infiltration of microglia (**E**), hemopoietic cells (**F**), and all cells (**G**), respectively. Magnified segments of the tiled image are shown for a central portion of the scaffold (**A_1_**–**D_1_**) and the outer portion (**A_2_**–**D_2_**). A machine learning algorithm was designed to determine the number of hemopoietic cells (**E**), microglia (**F**), and all cells (**G**), as well as the total area of the scaffold. There was no significant difference between the conditions. One-way ANOVA with Tukey’s multiple comparisons were used. Error bars represent standard error, individual data points are displayed for each bar.

The in vitro data demonstrate that TiSAMP is cell- and protein (ECM)-attractive as more can bind to the surface than the number of those on bare OPF surfaces. While enhancement of axon regeneration is an important goal for treatment of an SCI, any modifications (chemical or architectural) to the implanted polymer scaffolds should not lead to increased negative side effects. Foreign body reactions leading to inflammation and scarring have been a long-time concern for neuroprosthetic and biomaterial scientists [33,34,36,37]. The cell- and protein-attractive surface may lead to greater cell infiltration into the scaffold, resulting in enhanced inflammation or fibrosis/scarring. Immunostaining was performed with CD45 to label hemopoietic cells, Iba1 to label with microglia/macrophages, and the DAPI counterstain to label all cell nuclei to determine the infiltration of cells into the scaffold after transplantation (Figure 9A–D,A1–D1: magnified areas from the central scaffold; A2–D2: magnified areas from the outer scaffold). A machine learning approach was used to identify and quantify the cell populations in a quick and non-biased manner (details in Supplementary Figure S3). All animals implanted with OPF scaffolds had equal numbers of hemopoietic cells (Figure 9E and Supplementary Figure S1A), microglia/macrophages (Figure 9F and Supplementary Figure S1B), and total number of cells (Figure 9G and Supplementary Figure S1C), which were equally distributed throughout the lengths of the scaffolds. The average numbers of hemopoietic cells were 82.88 ± 21.37 cells/mm^2^ for untreated OPF, 50.12 ± 5.19 cells/mm^2^ for full TiSAMP, 79.11 ± 19.90 cells/mm^2^ for 30 × 30 TiSAMP, and 91.65 ± 33.74 cells/mm^2^ for the seven-channel scaffolds (Figure 9E). The numbers of microglia/macrophage were 73.78 ± 27.13 cells/mm^2^ for untreated OPF, 55.66 ± 23.96 cells/mm^2^ for full TiSAMP, 90.57 ± 27.25 cells/mm^2^ for 30 × 30 TiSAMP, and 120.4 ± 37.34cells/mm^2^ for the seven-channel scaffolds (Figure 9F). Interestingly, these immune cell types only consisted of a small proportion of the total cells, which averaged 4592.4 ± 221.0 cells/mm^2^ for untreated OPF, 3956.3 ± 166.4 cells/mm^2^ for full TiSAMP, 3558.6 ± 375.4 cells/mm^2^ for 30 × 30 TiSAMP, and 4588.5 ± 80.9 cells/mm^2^ for the seven-channel scaffolds (Figure 9G). These results show no statistical differences in the types and numbers of cells that interacted with these different scaffolds after implantation. 

### 2.4. Rolled Scaffolds Have Equivalent Deposits of Collagen, Laminin, and Fibronectin as Those in Multichannel Scaffolds

**Figure 10:**
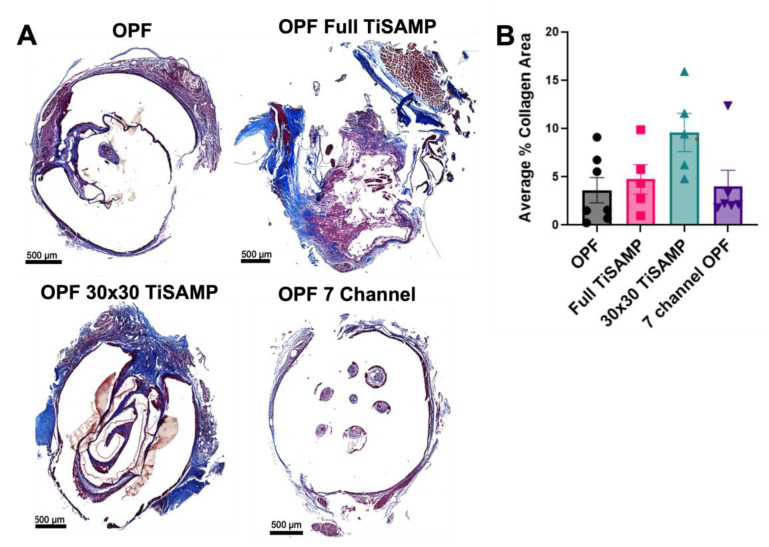
Fibrotic scarring following implantation of OPF scaffolds. (**A**) Implanted untreated OPF, Full TiSAMP, 30 × 30 TiSAMP, and seven-channel scaffolds were sectioned and stained at quarter lengths (¼, ½, and ¾ of the scaffold; center or ½ segment shown here) with Trichrome histological stain. The collagen component is indicated by blue staining, whereas the cytoplasm is pink. The dark blue stained parts represents areas containing excessive collagen deposits characteristic of fibrotic scarring, with light purple being the overlap of regular collagen deposits within the tissue. A machine learning algorithm was used to analyze the average percent area of collagen scarring at quarter lengths of the scaffold and then averaged (**B**). There was no significant difference between the conditions. One-way ANOVA with Tukey’s multiple comparisons were used. Error bars represent standard error, individual data points are displayed for each bar.

The extra open space could have been recognized as cavitation, or chemical processing could have been detected as an additional foreign object resulting in greater scarring. In addition, it has been noted that cells infiltrate into the scaffold and may contribute by laying down ECM on the TiSAMP surfaces. Trichrome staining was performed to determine the amount of collagen fibrotic scarring present in the rolled scaffolds compared to the seven-channel scaffolds (Figure 10A). A machine learning approach was employed to recognize the whole tissue and the scarred area from whole slide scans (details in Supplementary Figure S4). There was no statistical difference in the amount of collagen scar present across the scaffold types, with a roughly equal distribution across the length of the scaffold (Figure 10B and Supplementary Figure S2). The average percentages of scar area present were 3.62 ± 1.31% for untreated OPF, 4.73 ± 1.51% for full TiSAMP, 9.58 ± 1.98% for 30 × 30 TiSAMP, and 3.79 ± 1.69% for the seven-channel scaffolds (Figure 10B). A larger amount of the scar was observed on the outer surface of the rolled scaffolds than that which was internal. 

**Figure 11:**
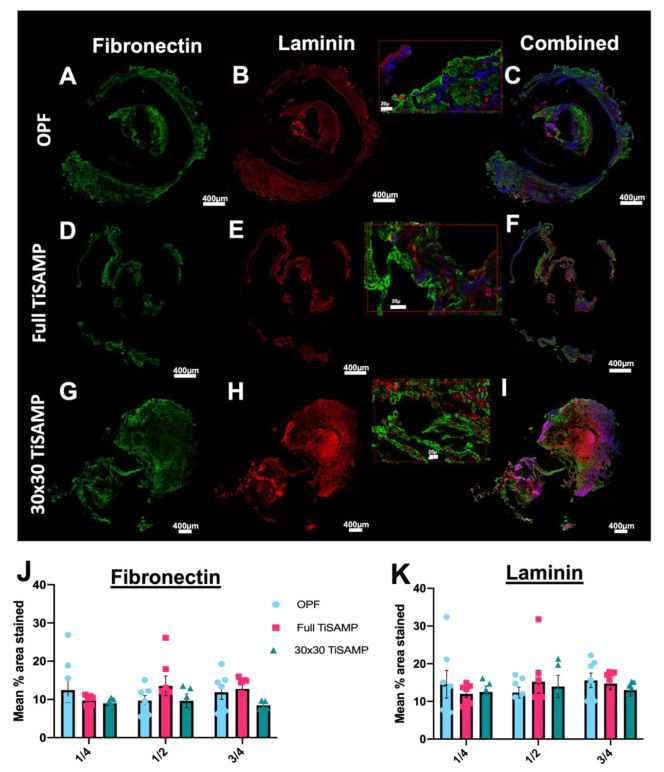
Fibronectin and laminin deposits by infiltrating cells following implantation of rolled OPF scaffolds. Quarter lengths (¼, ½, and ¾ of the scaffold; center or ½ segment shown here) of untreated rolled OPF scaffold (**A**–**C**), full TiSAMP OPF (**D**–**F**), and 30 × 30 TiSAMP (**G**–**I**) were stained for fibronectin and laminin. Magnified segment of the region of interest in the combined images (**C**,**F**,**I**) are shown in the red box. There was no significant difference in the mean percent area stained through the whole length of the scaffold (One-way ANOVA with Tukey’s multiple comparisons) of either fibronectin (**J**) or laminin (**K**). Error bars represent standard error, individual data points are displayed for each bar.

Since cultured cells can deposit ECM on the chemical pattern, which neurites can grow on and weave through (see Figure 7 and Figure 8), infiltrating cells may also contribute to the amount of ECM on the chemically processed scaffolds. Fibronectin (Figure 11A,D,G,J) and laminin (Figure 11B,E,H,K) are some of the major ECM types in the spinal cord. Equivalent amounts of fibronectin and laminin were detected across the lengths of the scaffolds, independent of whether they were chemically treated or untreated scaffolds (Figure 11C,F,I–K). The total contribution of ECM to the surface areas of the scaffolds was 10–20%. This indicates that the presence of the TiSAMP coat did not enhance or inhibit ECM deposition.

### 2.5. Implantation of Rolled Scaffolds Results in More Axon Growth through the Scaffold and Improved Hindlimb Function Compared to Those of Multichannel Scaffolds

**Figure 12:**
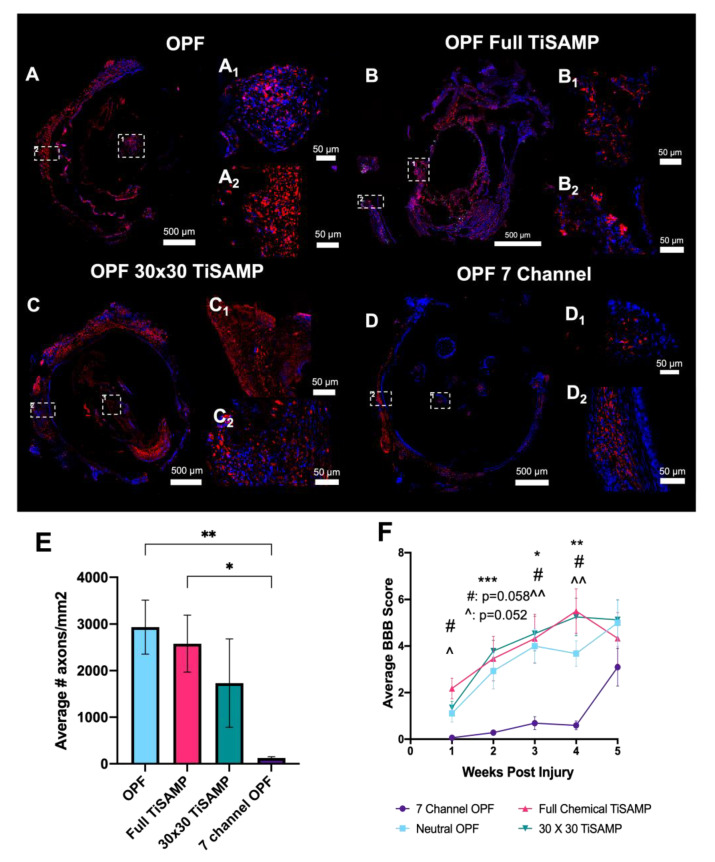
Axon outgrowth into rolled scaffold with or without TiSAMP compared to that of seven-channel scaffolds following transection and implantation into the T9 spinal cord. The implanted untreated OPF (**A**), full TiSAMP (**B**), 30 × 30 TiSAMP (**C**), and = seven-channel (**D**) scaffold were sectioned and stained at quarter lengths (¼, ½, and ¾ of the scaffold; center or ½ segment shown here) for β-tubulin (red) and DAPI (blue) to determine axon growth. Axons were determined via punctate staining positive for β-tubulin, whereas cells were β-tubulin positive with DAPI nuclei (likely phagocytosing cells) and were not counted as axons. Magnified segments of the tiled image are shown for a central portion of the scaffold (**A_1_**–**D_1_**) and the outer portion (**A_2_**–**D_2_**). The average number of axons growing through the rolled scaffold was greater than that of the seven-channel scaffolds. ((**E**) One-way ANOVA with Tukey’s multiple comparisons were used; error bars = SEM; degrees of significance are indicated as follows: *p* < 0.05 (*), *p* < 0.01 (**).) The average hindlimb function as measured in the BBB motor test also indicated that there was early and sustained hindlimb functional improvement when the rolled scaffold was implanted compared to that of the seven-channel scaffold ((**F**); purple line; Two-way ANOVA with Tukey’s multiple comparisons; degrees of significance are indicated as follows: *p* < 0.05 (*), *p* < 0.01 (**), *p* < 0.001 (***); * untreated rolled OPF compared to that of seven-channel OPF, # 30 × 30 TiSAMP OPF compared to that of seven-channel OPF, ^ full TiSAMP OPF compared to that of seven-channel OPF. ^^ level of significance but a different comparison.

Chemically treated, ridged OPF sheets (0.4 mm spaced ridges on one side and either no chemical treatment, full TiSAMP treatment, or patterned 30 × 30 TiSAMP treatment on the other side) were rolled into a single-channel OPF scaffold and implanted into rats following thoracic (T9) transection. The effect on axon outgrowth, inflammation, scarring, and behavioral recovery was compared between animals implanted with rolled OPF sheets and OPF in a multichannel (seven channel) design in the absence of any added ECM or Matrigel over a 5-week period. The immunostaining of axons with β-tubulin (Figure 12A–D) revealed that all three rolled scaffolds (untreated OPF contained 2936 ± 580.2 axons/mm^2^, *p* = 0.0091; full TiSAMP had 2579 ± 611.0 axons/mm^2^, *p* = 0.0243; 30 × 30 TiSAMP had 1733 ± 949.0 axons/mm^2^, *p* = *ns*) had greater axon counts than the seven-channel scaffold did (125.9 ± 26.92 axons/mm^2^, Figure 12E). Much of the β-tubulin staining in the seven-channel scaffold were cells that contained β-tubulin, possibly via the digestion of axonal/neuronal debris (axons are identified via punctate β-tubulin staining with no DAPI nuclei, whereas cells are indicated via β-tubulin staining containing DAPI nuclei; Figure 12D,D1: magnification of central scaffold and D2: magnification of outer scaffold). Although there is an increase in the number of axons in the scaffold, axons growing on the outside surface were still evident (Figure 12(A2–C2)). The rolled sheet design, with a more open surface area available in the same size scaffold as that of the seven-channel design, contained more axons per mm^2^, perhaps indicating a greater regenerative potential to make connections across the lesion. 

The BBB test is an open field test that measures hindlimb function based on a 21-point score, with 21 being normal function, a score of 8 representing that they are able to sweep with no weight support, and 0 being no function. To obtain the BBB score, we averaged two blinded scorers weekly over the 5-week period. The BBB scores matched the trend from the axons counts, where the animals implanted with either of the three rolled scaffolds had significantly greater scores from weeks 1 to 4 than those the animals implanted with the seven-channel scaffold (Figure 12F). The greatest difference in the BBB score was seen at week 3 (seven-channel scaffold score was 0.69 ± 0.28 vs. untreated OPF score of 4.00 ± 0.74, *p* = 0.015; full TiSAMP score of 4.32 ± 1.05, *p* = 0.048; 30 × 30 TiSAMP score of 4.54 ± 0.75, *p* = 0.006) and week 4 (seven-channel scaffold score was 0.59 ± 0.19 vs. untreated OPF score of 3.68 ± 0.55, *p* = 0.004; full TiSAMP score of 5.50 ± 0.96, *p* = 0.012; 30 × 30 TiSAMP score of 5.25 ± 0.81, *p* = 0.007). The animals implanted with the seven-channel scaffolds improved at week 5 to the point that the greater BBB scores of the rolled scaffolds were not significantly greater. These results suggest that a rolled scaffold design may facilitate the more rapid recovery of function perhaps via increasing the number of axons able to traverse the scaffold.

## 3. Discussion

Anatomical and functional recovery following an SCI requires a growth-permissive environment that guides axons across the injury to help bridge the gap and reform connections. Both short relay reconnections and the regeneration of long tracts can promote functional recovery [38]. One way to promote these reconnections is to provide appropriate growth environments with topographical cues, which can be achieved via the introduction of hydrogel scaffolds. This study demonstrates that chemically patterned TiSAMP OPF sheets can promote the alignment and outgrowth of cultured hMSCs, Schwann cells, and dispersed DRG neurons. The ECM matrix produced by these cells can also align on the patterned surface, and the use of multiple cell types can contribute different ECM types. In vivo, rats implanted with the open-spaced, rolled scaffold sheets with ridges had a greater degree of axon regrowth through the scaffold with improved early hindlimb recovery compared to that of the rats implanted with the multichannel scaffold without any increased inflammation, scarring, or ECM (laminin and fibronectin) deposition. Lastly, research on this topic is slowed down by in-depth histological analysis that is often required. We demonstrated a method by which machine learning utilizing neural networks can aid in the analysis of these data sets in a fast, non-bias mannered, allowing for the rapid characterization of implanted materials using an open-source software, QuPath (v0.3.2).

Attributes such as stiffness, permeability, swelling, strength and degradation kinetics are the important characteristics, which define the reaction of the surrounding tissue to the foreign implant [2]. The choice of the right biomaterials is, therefore, an important step in the subsequent development of a spinal cord scaffold. Hydrogels, which are water-absorbing cross-linked polymer networks, can simulate the mechanical and physical properties of the adjacent spinal cord [39]. However, hydrogels have lower protein adsorption, which reduces cell adhesion and structural support [40]. This problem is mediated via incorporating ECM proteins into the scaffold, adding adhesion sites, or via chemical processing, as demonstrated in this study. Biomaterials can be divided into two classes of being naturally and synthetically derived [41]. The natural biomaterials investigated for SCIs are collagen, hyaluronic acid, chitosan, gelatin, agarose, alginate, and fibrin [42,43,44,45,46,47,48,49]. Naturally derived biomaterials can have composition that is similar to that of the spinal cord and, generally, are thought to have good biocompatibility, biodegradability, low toxicity, intrinsic cellular interaction, and biological functionality [50]. However, synthetical polymers allow a wider range of customization via adjusting their composition and combining different polymers to create new ones [51]. Commonly used synthetic polymers are often composed of poly(lactic acid) (PLA), poly(lactic-co-glycolic acid) (PLGA), polyethylene glycol (PEG), and non-biodegradable hydrogels such as poly(2-hydroxyethyl methacrylate) (pHEMA) [52,53,54,55]. OPF scaffolds are hydrogels that have a PEG base and have a fumarate moeity [27]. By changing the ratios and concentrations of the cross-linker and polymer in the precursor solution, its biophysical properties can be fine-tuned to fit the demand of the target tissue [27]. In comparison to PLGA and poly(ε-caprolactone fumarate) (PCLF) scaffolds, OPF and positively charged OPF+ scaffolds have the most similar mechanical properties to that of spinal cord tissue [28].

Notably, the degree of axon regeneration, even without the aid of ECM molecules, cells, or other factors, was greater in the rolled scaffold sheet design than it was in the multichannel design. Previous studies have demonstrated that open-path polycaprolactone (PCL) scaffolds increase axon regrowth and have a smaller deficit size as compared to those of multichannel, tube, or cylinder designs [5]. However, multichannel scaffolds with an increased number of smaller channels can encourage greater axon regrowth as well [8]. This could partially be due to the larger channel scaffolds having larger fibrous rims. Our rolled scaffold design has a larger surface area than that of the multichannel design and is somewhat a hybrid of an open path and multichannel scaffold since the ridges do act as spacers between the layers and segment the open space. The use of rolled scaffolds, with their greater open spaces, did not result in any greater amount of fibrotic collagen scarring than the multichannel scaffolds did. This was an important consideration since previous studies with multichannel scaffolds demonstrated collagen-dense scarring in the area of the open channels and host interface surfaces [29,56]. Additional topographical cues are added via TiSAMP chemical patterning, which, in vitro helps to align and guide the growth of different cell types. This approach is most similar to the use of fibrils or grooves micro-architectures to promote elongation and alignment [13,57]. A PLGA scaffold designed as a cylindrical nanofibrous scaffold was demonstrated to help align cells and induce their proliferation in vitro, and after transplantation in a left lateral hemisection, it induced superior hindlimb function in lesion control animals in a BBB open field test [58]. Our study demonstrated a similar level of hindlimb function recovery when the rolled OPF scaffold sheet was implanted following a complete transection. Although, in the absence of added factors such as the ECM or cells, all three rolled scaffold types had a similar level of recovery and axon regrowth throughout the scaffold. However, all of the rolled scaffold sheets in this study have ridges that we have previously shown can guide neurites in vitro alone [13]. Future studies should explore whether adding aligned ECM or cells, which is aided by the TiSAMP patterning, can further enhance regeneration and recovery. 

MSCs are a popular cell type for use in CNS injuries and disease treatments. They can provide trophic support, modulate the immune system, aid tissue preservation, and promote angiogenesis [59,60]. On ridged surfaces, MSCs preferentially adhere to the ridges, instead of the adjacent flat area, thereby forming spheres. On flat OPF sheets, glass, or plastic, MSCs will have a stereotypical flat morphology. The addition of ECM protein coats did not aid in flat attachment to the ridged OPF scaffold. The MSCs produced collagen IV on the softer, spinal cord, such as the OPF material. When MSCs were grown on the OPF surface containing patterned TiSAMP, which was only a few molecular layers higher than the polymer spacing was, they attached and aligned on the scaffold without forming spheres. Taken together, this demonstrates the importance of the stiffness and physical structure of surfaces where the MSCs are expected to adhere to or engraft onto. When they are used for treatments for CNS injures and disorders, cells are injected into or on top of soft surfaces, such as the brain or spinal cord. However, many of the studies that are conducted to characterize MSCs in vitro use glass or plastic surfaces [61,62,63]. In this study, we demonstrate that cell characteristics can change depending on the surface morphology and stiffness. Gene expression changes, cell adhesion properties, and changes in proliferation have been previously described to be influenced by the structure and stiffness of the surface that the MSCs are grown on top of [64,65]. This scaffold, with or without TiSAMP, introduces a new way to investigate cells on surfaces that are more similar the biomechanical ones that they will be implanted on. In addition, the scaffold also presents a way to deliver pre-cultured cells and ECM to the spinal cord in future studies, while maximizing the surface area and promoting bidirectional orientation to bridge the gap after an injury. 

Schwann cells are another popular cell therapy choice for SCIs as they are the myelinating cell type of the peripheral nervous system and may encourage regeneration via the secretion of trophic factors, ECM proteins, and physical scaffolding [1,66,67]. Schwann cells are also affected by material stiffness and elasticity. When they are grown on softer surfaces (0.5–4 kPa), Schwann cells exhibit a rounded morphology and reduced motility and proliferation [68,69], whereas the stiffer surfaces of Schwann cells have a bipolar morphology with increased motility and proliferation. Surface stiffness can also have important functional relevance since during the development of peripheral nerves, the stiffness changes from around 6kPa when myelination starts to about 50 kPa during adulthood [70]. The results in this study show that Schwann cells grown on OPF scaffolds with TiSAMP attach and elongate on the chemical. The same stiffness-dependent effects of hMSCs were not seen. 

We also investigated the ability of Schwann cells to deposit ECM on the scaffolds. The amount of ECM produced by Schwann cells may be limited, and in the current study, they produced a little amount of fibronectin. Other cell types, such as fibroblasts, have been used to enhance the depositing of factors and ECM from Schwann cells [71]. In this study, we tested the capability of Schwann Cells to synergize ECM production with hMSCs. Human MSCs can produce various ECM components, such as fibronectin, laminin, collagen I and Tenascin-C [72], which can act as a substrate for Schwann cells. The co-culture of Schwann cells and hMSCs increased the fibronectin deposits by five-fold more than when they were cultured alone. Schwann cells were also able to add greater laminin components to the ECM in co-culture than those when the hMSCs were cultured alone. In addition, the ECM fibrils aligned with the direction of TiSAMP patterning. Certain ECM proteins are well known to assist nerve regeneration. During development, the expression of laminin and fibronectin coincides with the migration of neurons [73]. Laminin can guide axons and neurites, while fibronectin has a role in regulating cell differentiation, migration, and adhesion [74,75,76,77]. Various endogenous cells have been known to infiltrate scaffolds [32,57,78,79] and may contribute to their ECM composition. Since the TiSAMP chemical process aided in the deposition of ECM from cultured cells, infiltrating endogenous cell types may deposit ECM, which could aid regeneration. Following the trend of axon regrowth, all the implanted scaffold sheets had equal depositions of laminin and fibronectin. Since only a small proportion of the total cells were immune cells and, on average, the laminin and fibronectin areas were greater than the scar area was, ECM depositing by endogenous cells could have been beneficial to axon regrowth. This finding also provides parameters for the investigation of implanting exogenous ECMs, since endogenous cell infiltration can contribute to the overall composition. Therefore, strategies influencing the endogenous cell expression of inhibitory products in combination with biomaterials remains important. 

This study takes a novel approach to fabricating a scaffold for spinal cord repair via using a ridged, flat sheet design molded on a grooved Teflon plate, and then optimized using a new method to layer titanium dioxide and a self-assembled monolayer of phosphoric acid on hydrogel surfaces to increase the adhesion points on the scaffold. For implantation, the scaffold sheet was then rolled into the diameter of the spinal cord into a single tube OPF scaffold for implantation, or it could be used as flat sheets in a culture. However, alternative methods to fabricating scaffolds exist, such as electrospinning, self-assembly, solvent casting, and 3D bioprinting [80,81,82,83]. Three-dimensional bioprinting, in particular, has an interesting clinical application in creating patient-specific scaffolds using images captured via computer tomography or magnetic resonance imaging [84]. The materials used in this study can be adapted for other fabrication processes to aid in developing patient-specific constructions. We demonstrate that greater open spaces aid in axon regeneration, which could be achieved via using a spiral ridged design. The dimensions of these ridges and spaces, as well as anchoring enhancements using TiSAMP, can be adapted into other future designs.

## 4. Conclusions

Overall, this study demonstrates that OPF scaffolds fabricated as ridged sheets with chemical patterns can promote the alignment and outgrowth of cells in a bidirectional manner. This patterning also influences the directionality of produced factors, such as the ECM. When they are rolled and implanted into rats after spinal cord transection, the rolled scaffold sheets promote greater axon regeneration than the multichannel scaffolds do. We noted no additional immune cell infiltration or scarring. ECM depositing on the scaffold by endogenous cells may contribute to axon outgrowth. As the purpose of the study was to characterize the in vivo effects of the scaffold, this study was conducted up to 5 weeks post-injury. The first 4 weeks constituted the acute and subacute phases of the SCI, where inflammation, regeneration, and scar formation play important roles. However, it is important for the authors of future studies to explore the role of this scaffold during the chronic phase, particularly if it is functionalized with biologics. Future studies can use this scaffold design with the TiSAMP coating to deliver cells or functionalize the surface with ECMs. The ECM is comprised of fibrillar proteins and additional factors that are not only biocompatible and bioactive, but also reiterate the structural and compositional characteristics of the native environment [71,85]. Altering the types of cells, physical conditions, or the culture media make it possible to obtain a great diversity of specifically designed ECMs that are suitable for different implementations [71,85]. This study demonstrates that MSCs and Schwann cells can attach to TiSAMP and deposit ECM. This study provides a great regenerative medicine tool that can be used in vitro for cell characterization or in vivo for axon guidance, neuroprosthetics and devices production, or the future delivery of cells and patterned ECM.

## 5. Methods

### 5.1. OPF Sheet Fabrication

OPF was synthesized as previously characterized and described [13,27,28,86]. Briefly, a liquid OPF polymer composed of 1g of OPF macromere (16,246 g/mol), 950 µL deionized water, 0.3 g N-vinyl pyrrolidinone (NVP; Sigma Aldrich, St. Louis, MO, USA), and 0.05% (*w*/*w*) photoinitiator (Irgacure 2959; Ciba Specialty Chemicals, Basel, Switzerland) was made. A 0.08 mm thin OPF sheet with 0.1 mm high ridges was created using a Teflon mold, with a 0.08 mm spacer and grooves of 0.1 mm depth spaced 1 mm or 0.4 mm apart. The polymer was slowly pipetted onto the mold and covered with a glass plate. The mold was placed in a UV light oven for 1 h and cured overnight in the dark. The single-channel OPF scaffold was fabricated via the mold injection of the polymer, and then cast over a 1 mm wire. The single channel scaffold was then polymerized via exposure to an ultra-violet (UV) light (365 nm) for one hour and cured overnight. The scaffolds were cut into 2 mm lengths for transplantation. Similarly, for the seven-channel scaffold, the polymer was mold injected over 7 equally spaced 290 µm wires in a glass cylinder and cut into 2 mm lengths for transplantation.

### 5.2. OPF Polymer Chemical Micropatterning

Chemical processing was performed on the opposite side of the ridge surface of the OPF sheets (Figure 1) as previously described [17]. Briefly, in an inert atmosphere glovebox with low oxygen (0.3 ppm) and moisture (0.5 ppm) (OMNI-LAB Glove Box System, Vacuum Atmospheres Company, Hawthorne, CA, USA), a solution of 30 μL of titanium (IV) isopropoxide (CAS: 546-88-9) was mixed with 5 mL anhydrous toluene (CAS: 108-88-3). A hydrolysis reaction created a surface of TiO_2_ on the hydrogel for bonding with phosphonic acid. The phosphonic acid solution was created by mixing 1 mg of 12-(phosphonododecyl) phosphonic acid (CAS: 7450-59-1) with 5 mL anhydrous toluene. OPF sheets were hydrated and positions on glass slides, with the excess water blotted off. To create a pattern, a Kapton shadow mask with laser ablated slits of 30 μm and spaced with 30 μm material was placed on the OPF sheet. The sheet with the mask (or without the mask for fully coated sheets) was heated to 30 °C for 15 s and placed in the TiO_2_ for 30 s, followed by a rinse in toluene. The material was then heated to 30 °C for 30 s. The material was then placed into the phosphonic acid solution overnight, yielding a self-assembled monolayer of phosphonic acid (SAMP) on top of the TiO_2_ layer (TiSAMP). The scaffold was then rinsed in toluene, followed by isopropanol, and then distilled water. The mask was then removed and heated to 30 °C for 30 s. The sheets were then kept in double-distilled water until use.

### 5.3. Human MSC Isolation and Culture

Human MSCs (hMSCs) were isolated from adipose tissue biopsies, and then cultured, expanded, characterized, and cryopreserved by staff at the Mayo Clinic Human Cellular Therapy Laboratory as previously described in [87,88]. The release criteria included culture sterility, mycoplasma testing, and cytogenetic analysis, as well as cellular phenotyping, including CD90+, CD105+. CD73+, HLA Class I+, CD14+, CD44+, HLA DR, and CD45-. Cells were cultured in advance MEM media (Gibco, Thermo Fisher Scientific, Waltham, MA, USA) supplemented with human platelet lysate (5%; Lawson, Mill Creek, WA, USA), 100 U/mL Antibiotic-Antimycotic (Gibco Thermo Fisher Scientific, USA), 100 units/mL Glutamax (Gibco Thermo Fisher Scientific, USA), and 1000 USP units heparin (Novaplus, Lake Zurich, IL, USA) at 5% CO_2_ and 37 °C. 

### 5.4. Rat Schwann Cell Isolation and Culture

Rat Schwann cells were harvested from the sciatic nerve of 2–5-day-old Sprague Dawley pups as previously described [13,33,34,89,90]. The cells were cultured in DMEM/F12 (Gibco, Thermo Fisher Scientific, USA) containing 10% fetal bovine serum (FBS; Gibco, Thermo Fisher Scientific, USA), 100 U/mL Antibiotic-Antimycotic (Gibco Thermo Fisher Scientific, USA), 2 μM Forskolin (Sigma Aldrich, USA), and 10 ng/mL neuregulin-1 (R&D, Minneaplois, MN, USA) at 5% CO_2_ and 37 °C.

### 5.5. Co-Culture hMSC and Schwann Cells

For the co-culture experiments, first, the cells were grown alone as described above. Once they reached 80% confluence, they were cultured on scaffold sheets at a 1:1 ratio (75,000 cells per cell type). The co-culture medium of hMSCs and Schwann cells was composed of DMEM/F12 with L glutamine media (Gibco, Thermo Fisher Scientific, USA), Forskilin (2 µM; Sigma Aldrich, USA), Neuroregulin (10 ng/mL; R&D, USA), 10% FBS (Gibco, Thermo Fisher Scientific, USA), and 1% antibiotic/antimycotic (Gibco, Thermo Fisher Scientific, USA). 

### 5.6. Disassociated Rat Dorsal Root Ganglion Neurons

On day 15, embryonic Sprague Dawley rats were used to isolate the dorsal root ganglions (DRGs; previously described in [13,91,92]). Approximately 200 whole DRGs were pooled, and then dissociated via mechanical trituration in trypsin. Each scaffold was plated with 50,000 cells in MEM (Gibco, Thermo Fisher Scientific, USA) supplemented with 15% calf bovine serum (Hyclone, San Angelo, Texas, USA), 5 ng/mL nerve growth factor (Harlan Bioproducts, Indianapolis, IN, USA), 7 mg/mL glucose (Sigma Aldrich, USA), and 1.2 mM L-glutamine (Gibco, Thermo Fisher Scientific, USA). To obtain a pure culture of neurons, the cells were treated with 1 × 10^−5^ M 5-fluoro-2-deoxy-uridine and with 1 × 10^−5^ M uridine (Sigma Aldrich, USA) for 3 days. Two days after the treatment, neurons were fixed in 4% paraformaldehyde in PBS for 30 min and washed 3 times in PBS before the immunocytochemistry step.

### 5.7. OPF Preparation and Cell Seeding

Positively charged OPF (OPF+) sheets were created via the polymer containing 1 g of OPF macromere (16,246 g/mol), 650 µL deionized water, 0.3 g NVP (Sigma Aldrich, USA), and 0.05% (*w*/*w*) photoinitiator (Irgacure 2959; Ciba Specialty Chemicals, Basel, Switzerland), and 20% *w*/*w* 2-(methacryloyloxy)ethyl]-trimethylammonium chloride (MAETAC, Sigma-Aldrich, USA). The positively charged OPF+ sheets were only used in the study of the cells’ interactions with topography and stiffness, where chemical TiSAMP patterning was not introduced as a modification. A positive charge can help with cell attachment and outgrowth [86], but it may interfere with chemical patterning. OPF or OPF+ was hydrated in culture media and was pinned to sterile culture dishes containing SYLGARD 184 Silicone Elastomer (Dow Corning, Midland, MI, USA) to prevent the spontaneous rolling of the scaffold. The plates were sterilized after 20 min exposure to UV and multiple ethanol washes before pinning, while the scaffold sheets were sterilized via multiple isopropanol washes. OPF sheets were coated with either media only, poly-l-lysine (PLL; Sigma Aldrich, USA; 0.01%; 5 min), laminin (100 μg/mL; Sigma-Aldrich, USA), or rat plasma fibronectin (10 μg/mL) at 37 °C overnight. Fibronectin was isolated from rat serum using gelatin Sepharose affinity chromatography as previously described [18]. The excess ECM protein coat or PLL was removed after 3 washes with culture media specific to the cell type that would be plated. Once the cells were 70–80% confluent, 150,000 cells were seeded onto each OFP sheet for 7 days. Co-cultured cells (50:50) were plated with 75,000 cells of each type. Three sheets per condition were used. The OPF sheets with cells were fixed using 4% paraformaldehyde in phosphate-buffered saline for 20 min. Three (3) washes with PBS were performed to remove excess PFA, and then immunocytochemistry was performed.

### 5.8. Immunocytochemistry of Mincultured Cells on Scaffolds

Non-specific binding sites were blocked using a solution containing PBS, 10% normal donkey serum, and 0.3% Triton X-100 for 30 min. Then, scaffolds containing cells were incubated with rabbit α fibronectin (1:100; R184), mouse α laminin (1:100; 2E8; Developmental Studies Hybridoma Bank, USA [93]), and mouse α β-tubulin (1:300; Millipore Sigma, Burlington, MA, USA) overnight at 4 °C. Then, the samples were washed 3 times with PBS, and then incubated for 1 h with the donkey α mouse Cy3 (1:200; Millipore Sigma, USA), donkey α rabbit Cy5 (1:200; Millipore Sigma, USA), and phalloidin conjugated to Alexa 488 (1:100; Molecular Probes, Eugene, OR, USA) at room temperature. Then, the samples were washed 3 times in PBS before being placed on a slide and coverslip in SlowFade Gold containing DAPI (Invitrogen, Waltham, MA, USA). In the case of cells grown on coverslips, they were inverted onto a glass slide with SlowFade Gold containing DAPI (Invitrogen, USA) following the same procedure above.

### 5.9. Image Acquisition for Scaffold Sheets

Fluorescent images were acquired using an inverted fluorescence microscope Zeiss Axio Observer Z-1 with a motorized stage that had an Axiocam 503 camera mounted on it (Carl Zeiss, Oberkochen, Germany). Zen (v2, blue edition; Carl Zeiss, Germany) software was used to acquire and process the images. Z-stacks with approximately 5 slides were taken at 20×. Bright-field images of live cultures were taken on a Zeiss Axiovert Model 35 microscope with a Nikon CCD camera at 10× magnification (Carl Zeiss, Germany). On each OPF sheet or coverslip, 4 representative areas were taken in a non-biased manner by moving one field of view up or down from a central position and 2 fields of view across. Three to four different replicates were used for each condition (three to four scaffolds or coverslips).

### 5.10. Analysis of Laminin and Fibronectin Deposit by Schwann Cells and MSCs on Scaffolds in Culture 

Fluorescent images were analyzed using ImageJ (v1.53) software (National Institutes of Health, Bethesda, MD, USA). Fluorescence intensity was measured as previously described [13]. Briefly, images type was set to 8-bit, and the mean grey value of laminin and fibronectin was measured. The mean grey value of laminin and fibronectin was then normalized to the mean grey value of DAPI staining per cell. One-way ANOVA with Tukey’s multiple comparisons was performed to determine statistical significance. Four representative areas were taken in a non-biased manner by moving one field of view up or down from a central position and 2 fields of view across. Four different replicates were used for each condition (four scaffolds or coverslips).

### 5.11. Scaffold Preparation and Surgical Implantation

OPF sheets and single-channel scaffolds were sterilized with a serial dilution of isopropanol. Two (2) mm by six mm OPF sheets were rolled and placed into the single channel scaffold. Female Sprague Dawley rats (230–300 g; Envigo, Indianapolis, IN, USA) received a laminectomy at level T9, and complete spinal cord transection was performed. The scaffold was placed in the transected area, and all the muscles were sutured closed. The rats were divided into 4 groups that were implanted with either untreated rolled OPF sheets (OPF; n = 7), fully coated TiSAMP OPF sheets (full TiSAMP; n = 7), patterned TiSAMP sheets with 30 μm thick chemical and 30 μm thick polymer spacing (30 × 30 TiSAMP; n = 7), or seven-channel OPF (n = 8) scaffolds. The animals were cared for daily by veterinarians and other staff experienced in the care of rats with spinal cord injuries. All procedures were approved by the Mayo Clinic Institutional Animal Care and Use Committee, and all guidelines were followed in accordance with the National Institute of Health, Institute for Laboratory Animal Research, the United States Public Health Services Policy, on the Humane Care and Use of Laboratory Animals, and ARRIVE. 

### 5.12. Basso, Beattie, and Bresnahan (BBB) Locomotor Score

The BBB open field locomotor test [34,94] was conducted weekly following the injuries to access hindlimb function. Two blinded observers rated aspects of the hind-limb function, which was scored on a 21-point scale. BBB scores were averaged between the observers, as well as the left- and right-side scores. Two-way ANOVA with Tukey’s multiple comparisons was used to determine statistical differences between the groups. 

### 5.13. Tissue Preparation and Immunohistochemistry

The animals were euthanized using anesthesia and fixed via transcardial perfusion with 4% paraformaldehyde in PBS. The spinal column was removed en bloc and post-fixed for 2 days at 4 °C. The spinal cords were dissected out, and a 1 cm segment with scaffold (T9) in the center was embedded in paraffin. Transverse sections 10 μm thick were cut using a Reichert-Jung Biocut microtome (Leica, Bannockburn, IL, USA). 

The tissue sections were deparaffinized using serial dilutions of xylene and ethanol. Antigen retrieval was performed by immersing the slides in 1 mM EDTA in PBS (pH 8.0), and then placing them in a streamer for 30 min. The tissue was then permeabilized with PBS containing 0.3% Triton X-100. Then, blocking solution containing PBS with 0.3% Triton X-100 and 10% normal donkey serum was placed on the tissue for 30 min to block non-specific binding sites. Then, the sections were incubated using the primary antibodies, rabbit α fibronectin (1:100; R184), mouse α laminin (1:100; 2E8; Developmental Studies Hybridoma Bank, USA [93]), mouse α β-tubulin (1:300; Millipore Sigma, USA), mouse α CD45 (1:50; Agilent DAKO, Santa Clara, CA, USA), and rabbit α Iba-1 (1:800; Wako FUJI Film, Richmond, VA, USA), overnight at 4 °C. The sections were then serially washed with PBS containing 0.1% Triton X-100. Secondary antibodies, donkey α mouse Cy3 (1:200; Jackson ImmunoResearch Laboratories, West Grove, PA, USA) and donkey α rabbit Alexa 647 (1:200; Jackson ImmunoResearch Laboratories, USA), were then placed on the sections for 1 h at room temperature. The secondary antibodies were washed off using PBS, and then cover slipped with SlowFade Gold containing DAPI (Invitrogen, USA).

### 5.14. Trichrome Staining 

Trichrome staining was performed using a Masson Trichrome Staining Kit (Richard-Allan Scientific, San Diego, CA, USA). The samples were first deparaffinized using serial dilutions of xylene and ethanol. The sections were then placed in Bouin’s fluid for 1 h at 56 °C. After a 5 min rinse in tap water, the slides were placed in working Weigert’s solution for 10 min. Following a 5 min rinse in tap water, the slides were then placed in Biebrich Scarlet solution for 5 min. The slides were then rinsed in distilled water for 30 s, followed by immersion into Phosphotungstic-Phosphomolybdic Acid for 5 min and Aniline Blue for 5 min. Then, samples were then washed in 1% Acetic acid for 1 min and rinsed in distilled water for 30 s. Lastly, the samples were then washed in 100% ethanol twice and xylene 3 times for I minute each. The slides were then mounted with a mounting medium (Richard-Allan Scientific, San Diego, CA, USA).

### 5.15. Image Acquisition

Immunofluorescent imaging was performed using a Zeiss LSM 780 Confocal Microscope (Carl Zeiss, Germany) with a motorized stage using Zeiss Zen (v2, blue edition) software. Whole transverse sections were imaged using a Plan Apochromat 20x/0.8 M27 objective. Light microscopy was performed using a Zeiss Axio Scanner Z1 (Carl Zeiss, Germany) with the Zen (v2, Blue edition) software. All sections on the slide were scanned using the automated tissue detector feature in Zen (v2, blue edition) software. 

### 5.16. Axon Count

Axons were identified via characteristic punctate staining with β-III tubulin by a blinded investigator. Axons were counted on the whole transverse section at quarter lengths throughout the scaffold. Tiled 20× images were loaded into the QuPath (v0.3.2) Software [29], and a grid was overlayed on the image. Using the point tool, axons were manually counted by a blinded investigator. was Axons were counted if they were in the quadrant or overlapped on the top horizontal or right vertical boundary. The area of the scaffold was measured by tracing the scaffold boundary. The axons counts were then averaged throughout the quarter lengths of the scaffold and normalized to area for each rat. One-way ANOVA with Tukey’s multiple comparisons was used to determine differences between the groups, and the errors are represented as ±SEM. 

### 5.17. Quantification of Laminin and Fibronectin Components of the ECM in Implanted Scaffolds 

A blinded investigator used ImageJ (National Institutes of Health, USA) to calculate the laminin and fibronectin stained areas in the fluorescent images throughout the quarter length of the scaffold. First, the region of interest around the scaffold was marked. The image was changed to 8-bit, and a threshold was set to select the staining in each channel. A percent area was calculated from the area stained to the area of the region of interest. Data represent quarter lengths or averages throughout the scaffold regions for each rat. One-way ANOVA was performed to determine differences between the conditions with the error represented as ± SEM.

### 5.18. Machine Learning Analysis of Immune Cell Infiltration and Fibrotic Scarring 

For the image analysis of immune cell infiltration (CD45+ cells and Iba-1+ cells), a neural network was implemented using QuPath (v0.3.2).software, which represents a fast, unbiased, and reproducible way to analyze microscopy images (Supplementary Figure S3). To identify the region of interest, a simple threshold pixel classifier was created to annotate the tissue section. Next, we configured a watershed cell detection algorithm to detect cells based on the intensity values of the DAPI channel. To enable more accurate classification, additional features were calculated for each cell, including the standard deviation of stain intensity for each stain, circularity, and perimeter. In the last step, we trained two separate networks on a large subset of training data produced by manually annotating cells for each antibody. Both neural networks were later combined into a composite classifier that could differentiate between CD45+ cells, Iba-1+-positive cells, cells positive for both markers, and cells negative for both markers. The data are represented as an average of all the quarter lengths throughout the scaffold per rat or counts at each quarter length for each rat. One-way or Two-way ANOVA with Tukey’s multiple comparisons was used where appropriate. 

For the image analysis of the fibrotic collagen scar area, a color deconvolution-based technique was implemented in QuPath (v0.3.2) for bright-field images (Supplementary Figure S4). First, the stain vectors were estimated using pixel values to separate out Analine blue stain from the Biebrich Scarlet Stain. A simple pixel threshold classifier was used (as explained for immunofluroscent images) to detect the tissue area. Then, color deconvolution was performed to calculate the percent area of scarred tissue (more detailed methodology is available in the Supplementary Information). Measurements for the whole tissue area and scar area were exported, and percentages for fibrotic, scarred area were obtained by dividing scarred tissue area by the whole tissue area. One-way or Two-way ANOVA with Tukey’s multiple comparisons was used where appropriate. 

### 5.19. Statistical Analysis 

All data are reported as the mean ± standard error of the mean (SEM), and analysis was conducted using GraphPad Prism (v9, GraphPad Software Inc.). Statistical significance was calculated using One-way or Two-way ANOVA with Tukey’s multiple comparisons as appropriate. One-way ANOVA was used to determine how one factor affects a response to a variable in a group of 3 or more. Two-way ANOVA was used to determine how 2 factors affect a variable and the interaction of the factors on the variable. All experiments were determined to meet the assumptions of ANOVA testing, with the data having normal variance and distribution. For in vitro experiments, 3 groups were compared (hMSCs, Schwann cells, and combined), while for in vivo experiments, 4 groups were compared (OPF, full TiSAMP, 30 × 30 TiSAMP, and seven-channel scaffolds). Each animal group had 7–8 rats. For the histological analysis of scaffolds, tiled images at ¼, ½, and ¾ lengths were taken and either analyzed separately or averaged. A *p*-value of 0.05 was used to determine statistical significance. The graphical representation of the *p* value is represented as * *p* < 0.05, ** *p* < 0.01, *** *p* < 0.001, or **** *p* < 0.0001, unless otherwise stated in the figure caption.

## Data Availability

All data are available on reasonable request. Contact Nicolas Madigan (madigan.nicolas@mayo.edu) for requests.

## Supplementary Materials

The following supporting information can be downloaded at: https://www.mdpi.com/article/10.3390/ijms241210250/s1, [95,96,97,98,99,100].
