# Supplementary material for: Open-Spaced Ridged Hydrogel Scaffolds Containing TiO2-Self-Assembled Monolayer of Phosphonates Promote Regeneration and Recovery Following Spinal Cord Injury
Source: Int J Mol Sci. 2023 Jun 16;24(12):10250. doi: 10.3390/ijms241210250 (PMC10299197; doi:10.3390/ijms241210250)
Supplement: Supplementary file 1 [file ijms-24-10250-s001.zip › ijms-2296916-supplementary.pdf]

Supplementary Information:

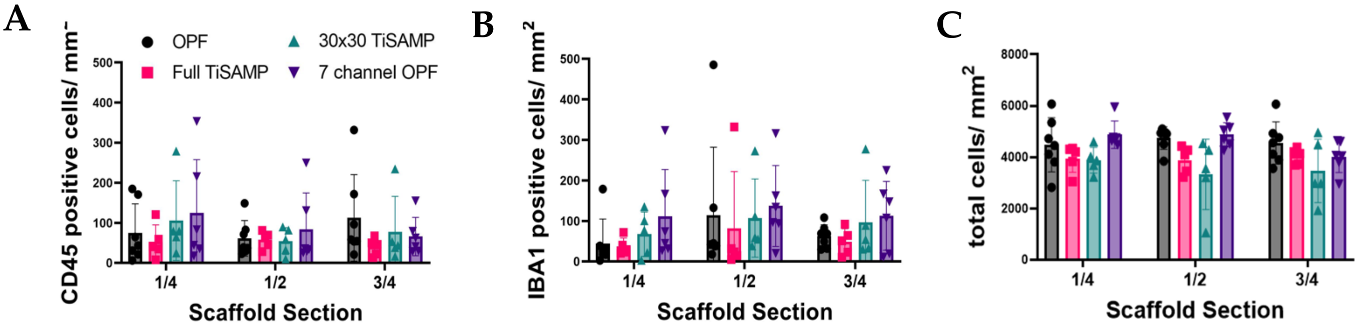

Figure S1. *Infiltration of immune cells per segment into the implanted OPF scaffolds. Implanted untreated OPF, Full TiSAMP, 30x30 TiSAMP, and 7 channel scaffold were sectioned and stained at quarter lengths (¼, ½, and ¾ of the scaffold) and then analyzed using A machine learning algorithm was designed to determine the number of CD45+ hemopoietic cells (A), Iba-1+ microglia (B), and DAPI for all cell nuclei (C). There was no significant difference between the conditions. One-Way ANOVA with Tukey's multiple comparisons.*

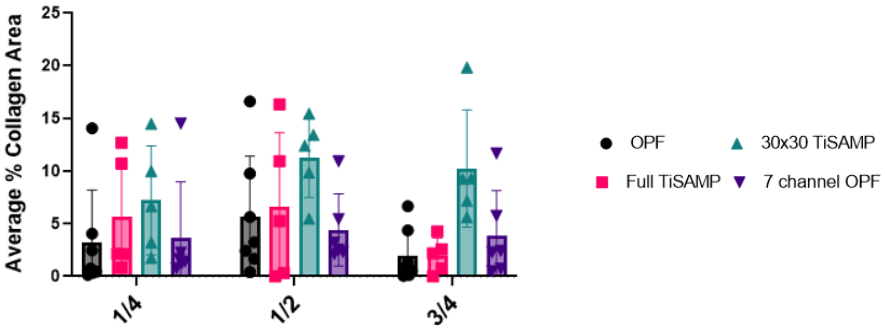

Figure S2. *Fibrotic scarring per segment of scaffold following implantation. Untreated OPF, Full TiSAMP, 30x30 TiSAMP, and 7 channel scaffolds were implanted and then sectioned 5 weeks post injury. Quarter lengths (¼, ½, and ¾) of the scaffold were stained with Trichrome histological stain. A machine learning algorithm was used to analyze the average percent area of collagen scarring at quarter lengths of the scaffold. There was no significant difference between the conditions. One-Way ANOVA with Tukey's multiple comparisons.*

Machine learning and neural networks for quantification of immune cell populations and fibrotic scar area

Histological and immunohistological analysis of tissue samples is a staple in neuroscience and regenerative medicine. After therapeutic intervention, such as after implantation of biomaterials, many aspects such as axon regeneration, immune infiltration, scarring, infiltration of endogenous cells, vascularization, and tissue preservation need to be measured [1-5]. The in-depth analysis of these parameters using manual methods is time consuming. In the area of pathology and diagnostics, there has been a rise of digital pathology to use artificial intelligence and machine learning to inform clinical research and clinical decision making [6,7]. Many new proprietary software have become available to establish pathology workflows informed by machine learning, however the uptake in basic science and pre-clinical research has been slower. In addition, these proprietary software can be costly for many research laboratories budgets [8]. In this paper we demonstrate how a free open-source software, QuPath, can be used to develop machine learning algorithms to analyze immunostained and histologically stained samples to determine level of immune cell infiltration and fibrotic scarring after scaffold implantation.

QuPath has 3 types of supervised machine learning algorithms built in: random forest classifiers, K-nearest neighbor classifier, and neural networks. Random forest classifiers are computationally less extensive and typically tabular results can be produced without extensive configuration of the classifier by the user [9,10]. Random forest classifiers depend on network of decision trees that are together are used to determine an outcome. This process has the disadvantage that it can overfit the data. K-nearest neighbor classifiers are one of the earliest algorithms used in medical research for pattern and object recognition [10]. K-nearest neighbor uses nonparametric clustering where the classification is based on the number of k neighbors. The disadvantage of K-nearest neighbor is that processing time becomes longer for larger number of classification and data, therefore having limited scalability. Neural networks are useful when processing large number of features and allows for more complicated classification tasks [9]. Neural networks try to mimic

the brain, where a network of artificial neurons use inputs to assign mathematical weights to predict outcomes. If not monitored, like other approaches, neural networks can overfit data. However, it has been argued that using neural networks for image processing to make clinical decisions was more accurate and required less input than random forest classifiers [11].

Quantification of histology can be time consuming and acts as a barrier for production of in-depth quantitively studies of regenerative medicine approaches in a timely manner. In this study, we used neural networks to quantify immune cell infiltration (CD45+ and Iba-1+ cells) and fibrotic scar area (using Trichrome staining) through quarter lengths of implanted scaffolds. Once trained, the application of the learned algorithm allows for fast, non-bias quantification of large image sets.

Neural Networks for quantification of immune cell infiltration into implanted scaffolds form micrographs of immunostained fluorescence images

For the quantitative analysis of immune cell infiltration in each scaffold, we implemented a neural network inside the open-source image analysis software QuPath. First, we created a new QuPath project by loading all the immunostained fluorescence images and setting their image type to "Fluorescent" inside QuPath to facilitate the following differentiation of the different channels. The channel names were set to DAPI, Iba1 and CD45. Next, we created a pixel classification thresholder which outlines the borders of the tissue sample per slide based on the DAPI channel pixel intensities. This allowed us to create accurate regions-of-interest for the later classifiers. We set the minimum object size to 1000 μm and the minimum hole size to 15000 μm to produce the most accurate results when measuring the area. To now obtain cell counts for the tissue regions, we implemented a cell detection watershed classifier. This classifier created

outlines for each nuclei, using again the pixel intensities of the DAPI channel with a minimum area of 10 μm, maximum area of 150 μm and a pixel intensity threshold of 35.

As the performance of machine learning classifiers can be increased by careful selection of fitting features, we calculated additional features for each cell to obtain more accurate results. We considered two classes of features: shape and intensity. Shape features included area, length, circularity and solidity, while intensity features included the mean, minimum, maximum, standard deviation and median values of pixel intensities of the CD45 and Iba-1 channel of the previously detected cells.

To create a training set for the machine learning classifier, an investigator manually labeled cells on randomly chosen images as positive for CD45, positive for Iba-1, positive for both or negative. We then implemented two neural networks inside of QuPath, one for the detection and classification of CD45+ cells and one for Iba-1+ cells. The neural networks had the same architecture with 3 layers and 12 neurons in the hidden layer. We trained them for 1000 iterations and the RPROP (resilient backpropagation) optimization algorithm for achieve increased performance on the gradient descent.

After training we combined both neural networks to create a composite classifier which is capable to differentiate between cells positive for CD45, Iba-1, both antibodies or negative for both.

To further automate the whole analysis process, we combined all the steps described above in a computer script written in the scripting language groovy. Executing this script directly inside QuPaths build-in Script Editor allowed us to pipeline the whole process and further reduce the chance of introducing bias into the process.

Lastly, all measurements were exported as a csv file for statistical analysis.

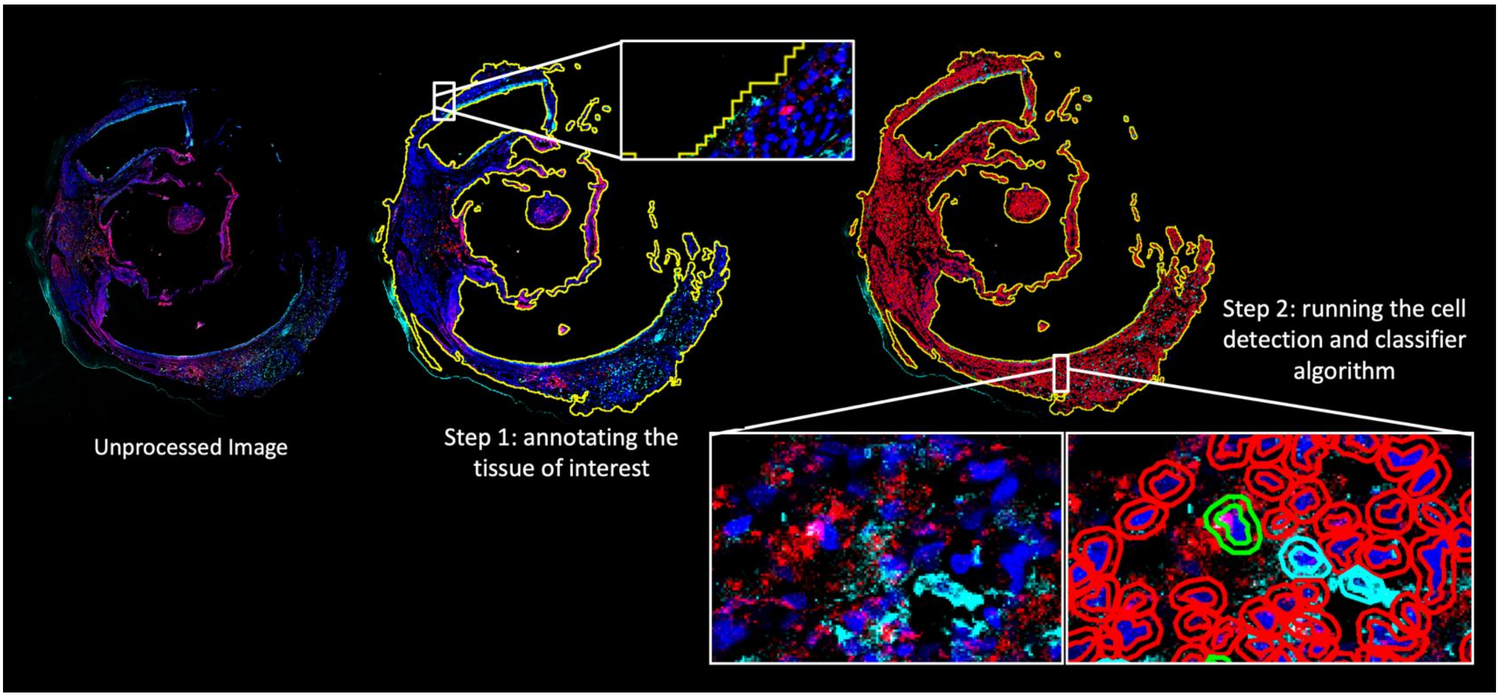

Figure S3. ***Machine learning identification of immune cell infiltration into the scaffold following implantation.*** Tissue sections containing implanted scaffolds were stained at quarter lengths with CD45 (cyan) and Iba-1 (red) antibodies. First the tissue of interest was annotated using a threshold algorithm to identify the borders of the tissue of interest. Training examples were created using single cell annotations as well as region wide annotations combined with a filter to only include objects identified as cells by the previous cell detection step. The number of training cells was approximately 20,000. This cell detection and classifier algorithm was ran on the whole data set to identify negative, positive, double positive labelled cells. Measurements for tissue area, total cell count, number of CD45+ cells, Iba-1+ cells, and cells positive for both antibodies was then exported for statistical analysis in Prism GraphPad Software.

Color deconvolution based fibrotic scar differentiation from whole slide micrographs of brightfield images

For the analysis of the trichrome stained brightfield images we created a new QuPath project, again loading the images and setting their image type to "Brightfield" this time inside QuPath. We first performed a color deconvolution step by estimating the stain vectors of the images. For this step, representative regions of the images were selected which included stained tissue as well as background. This allowed to separate the red and blue stains more precise to differentiate the tissue later. Next, we created an outline of the tissue based on a pixel threshold classifier, similar to the procedure for the immunofluorescent images. The channel for the pixel threshold classifier was set to be the average pixel values over all the channels.

For the classification of scarred tissue, we created a second pixel threshold classifier to only detect fibrotic and scarred area based on the Aniline Blue stain. This stain labels collagen, which is expressed in excessive amounts in fibrotic tissue. Therefore, the intensity of the Aniline Blue stain correlates with the density of collagen in the tissue. The threshold for scar detection was internally validated by comparing the results with other images of trichrome-stained tissue and empirically set to 0.8.

The two classifiers in combination were used to first label the tissue of interest and then classify the scarred portion of the tissue. All the steps were again combined into a computer script which allows faster execution and reproducible results.

Final measurements were exported and percentages for fibrotic and scarred area were obtained by dividing scarred tissue area by whole tissue area.

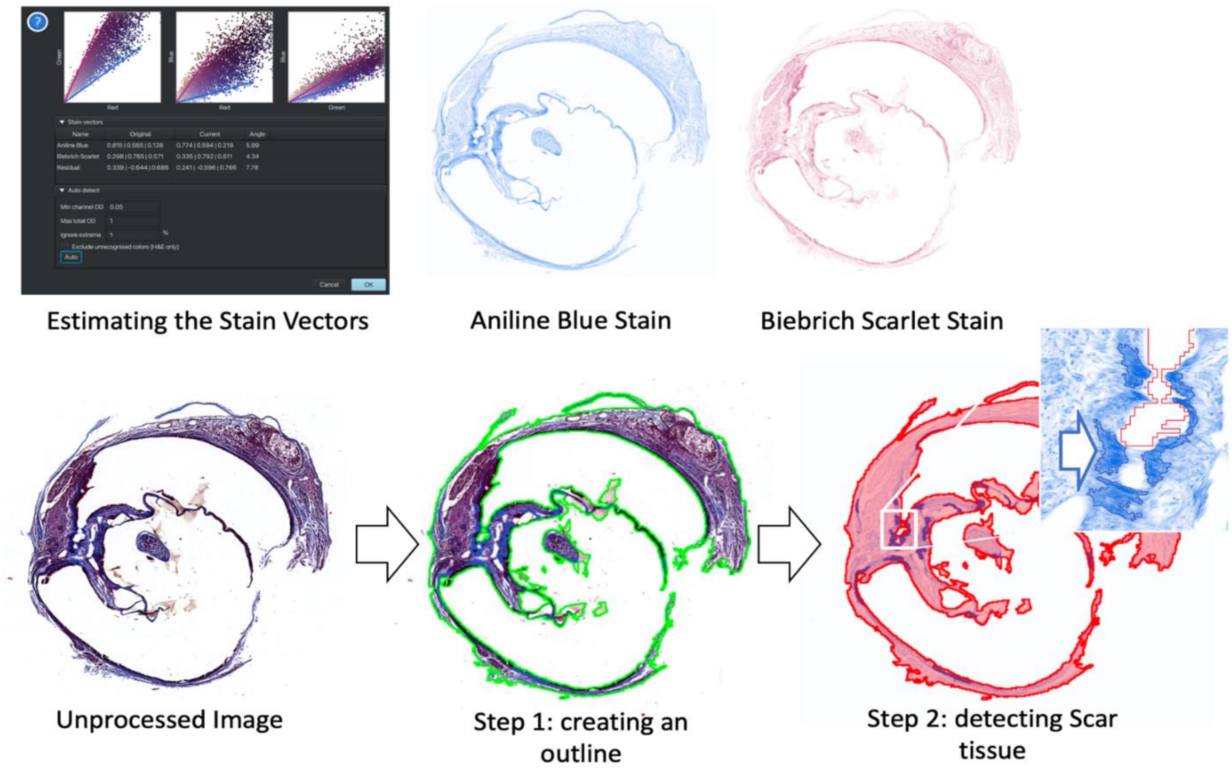

Figure S4. ***Color deconvolution based analysis of fibrotic scar area from Trichrome stained samples.*** Scaffold tissue samples at quarter lengths through the scaffold were stained with Trichrome histological staining method. Brightfeild scans of whole slides were taken and saved as individual files. To accurately distinguish the Aniline Blue stain (blue) from the Biebrich Scarlet stain (red), the stain vectors were estimated using pixel value representations. After this color deconvolution, a pixel threshold classifier was used to identify the boundaries of the tissue. The scar tissue was trained on a second pixel classifier designed to identify the excessive depositing of collagen stained by the Aniline Blue which is identified by the darker blue staining (arrows in step 2) than the ubiquitous collagen present in the tissue. The measurements for the whole tissue area and scar area were exported and percentages were calculated by dividing the scar area by the total area. Statistical analysis was performed in Prism GraphPad software.

References

1. Siddiqui, A.M., *et al.* Defining spatial relationships between spinal cord axons and blood vessels in hydrogel scaffolds. *Tissue Engineering Part A* **epub ahead of print**(2021).

2. Siddiqui, A.M., *et al.* Promoting neuronal outgrowth using ridged scaffolds coated with extracellular matrix proteins. *Biomedicines* **9**, 479 (2021).
3. Siddiqui, A.M., *et al.* Newly regenerated axons via scaffolds promote sub-lesional reorganization and motor recovery with epidural electrical stimulation. *npj Regenerative Medicine* **6**, 66 (2021).
4. Chen, B.K., *et al.* GDNF Schwann cells in hydrogel scaffolds promote regional axon regeneration, remyelination and functional improvement after spinal cord transection in rats. *Journal of tissue engineering and regenerative medicine* **12**, e398-e407 (2018).
5. Hakim, J.S., *et al.* Combinatorial Tissue Engineering Partially Restores Function after Spinal Cord Injury. *Journal of tissue engineering and regenerative medicine* (2019).
6. Bera, K., Schalper, K.A., Rimm, D.L., Velcheti, V. & Madabhushi, A. Artificial intelligence in digital pathology — new tools for diagnosis and precision oncology. *Nature Reviews Clinical Oncology* **16**, 703-715 (2019).
7. Signaevsky, M., *et al.* Artificial intelligence in neuropathology: deep learning-based assessment of tauopathy. *Laboratory Investigation* **99**, 1019-1029 (2019).
8. Lujan, G., *et al.* Dissecting the Business Case for Adoption and Implementation of Digital Pathology: A White Paper from the Digital Pathology Association. *J Pathol Inform* **12**, 17-17 (2021).
9. Doupe, P., Faghmous, J. & Basu, S. Machine Learning for Health Services Researchers. *Value Health* **22**, 808-815 (2019).
10. Rashidi, H.H., Tran, N.K., Betts, E.V., Howell, L.P. & Green, R. Artificial Intelligence and Machine Learning in Pathology: The Present Landscape of Supervised Methods. *Acad Pathol* **6**, 2374289519873088 (2019).
11. Mao, J., *et al.* Automated diagnosis and quantitative analysis of plus disease in retinopathy of prematurity based on deep convolutional neural networks. *Acta ophthalmologica* **98**, e339-e345 (2020).
